# Neuron–Microglia Contacts Govern the PGE_2_ Tolerance through TLR4-Mediated de Novo Protein Synthesis

**DOI:** 10.3390/biomedicines10020419

**Published:** 2022-02-10

**Authors:** Hsing-Chun Kuo, Kam-Fai Lee, Shiou-Lan Chen, Shu-Chen Chiu, Li-Ya Lee, Wan-Ping Chen, Chin-Chu Chen, Chun-Hsien Chu

**Affiliations:** 1 Division of Basic Medical Sciences, Department of Nursing, Chang Gung University of Science and Technology, Chiayi 61363, Taiwan; guscsi@gmail.com; 2 Chang Gung Memorial Hospital, Chiayi 61363, Taiwan; 3 Research Center for Food and Cosmetic Safety, College of Human Ecology, Chang Gung University of Science and Technology, Taoyuan 33303, Taiwan; 4 Chronic Diseases and Health Promotion Research Center, Chang Gung University of Science and Technology, Chiayi 61363, Taiwan; 5 Department of Pathology, Chang Gung Memorial Hospital, Chiayi 61363, Taiwan; lkf2002@cgmh.org.tw; 6 Graduate Institute of Medicine, College of Medicine, Kaohsiung Medical University (KMU), Kaohsiung 80708, Taiwan; shioulan@kmu.edu.tw; 7 National Laboratory Animal Center (NLAC), NARLabs, Tainan 74147, Taiwan; cscsc@narlabs.org.tw; 8 Grape King Biotechnology Inc (Grape King Bio Ltd.), Zhong-Li, Taoyuan 32542, Taiwan; ly.lee@grapeking.com.tw (L.-Y.L.); wp.chen@grapeking.com.tw (W.-P.C.); gkbioeng@grapeking.com.tw (C.-C.C.); 9 Institute of Molecular Medicine, College of Medicine, National Cheng Kung University, Tainan 70456, Taiwan

**Keywords:** PGE_2_, COX-2, microglia, endotoxin tolerance, cell–cell contacts, TLR4, de novo protein synthesis, LPS binding protein, innate immune memory, brain immunity

## Abstract

Cellular and molecular mechanisms of the peripheral immune system (e.g., macrophage and monocyte) in programming endotoxin tolerance (ET) have been well studied. However, regulatory mechanism in development of brain immune tolerance remains unclear. The inducible COX-2/PGE_2_ axis in microglia, the primary innate immune cells of the brain, is a pivotal feature in causing inflammation and neuronal injury, both in acute excitotoxic insults and chronic neurodegenerative diseases. This present study investigated the regulatory mechanism of PGE_2_ tolerance in microglia. Multiple reconstituted primary brain cells cultures, including neuron–glial (NG), mixed glial (MG), neuron-enriched, and microglia-enriched cultures, were performed and consequently applied to a treatment regimen for ET induction. Our results revealed that the levels of COX-2 mRNA and supernatant PGE_2_ in NG cultures, but not in microglia-enriched and MG cultures, were drastically reduced in response to the ET challenge, suggesting that the presence of neurons, rather than astroglia, is required for PGE_2_ tolerance in microglia. Furthermore, our data showed that neural contact, instead of its soluble factors, is sufficient for developing microglial PGE_2_ tolerance. Simultaneously, this finding determined how neurons regulated microglial PGE_2_ tolerance. Moreover, by inhibiting TLR4 activation and de novo protein synthesis by LPS-binding protein (LBP) manipulation and cycloheximide, our data showed that the TLR4 signal and de novo protein synthesis are necessary for microglia to develop PGE_2_ tolerance in NG cells under the ET challenge. Altogether, our findings demonstrated that neuron–microglia contacts are indispensable in emerging PGE_2_ tolerance through the regulation of TLR4-mediated de novo protein synthesis.

## 1. Introduction

Microglia, the primary innate immune cells of the brain, maintain the central nervous system (CNS) homeostasis at physiological conditions [1,2]. With their high mobility, microglia survey, and guard brain microenvironment (surveillance), they can regulate normal development, growth, connection, and functions of the neurons for a lifetime [3]. In response to immune challenge, microglia, as the first defense and inflammatory responder, secrete a wide spectrum and various immunoregulatory factors to protect the neurons against invading pathogens [4]. At the end of the inflammatory process, microglia are back to the status of immune resolution [5]. Conversely, the unresolved inflammation caused by overactivated microglia further damages neurons [6]. However, the immunosuppressive mechanism of microglia in resolving inflammation remains unclear [7].

Recently, many studies have demonstrated the capability of microglia in developing innate immune memory to either enhance (trained immunity) or suppress (immune tolerance) subsequent immune responses [8,9]. In response to the lipopolysaccharide (LPS, glycolipid of the outer cell membrane of Gram-negative bacteria) challenge, activated microglia immediately produce superoxide and TNF-α, followed by the production of IL-1β, nitrite (NO), prostaglandin E2 (PGE_2_), and IL-6 at 24 h [10]. Subsequently, anti-inflammatory cytokines, such as IL-10, are secreted by microglia for neuroinflammation resolution. Under recurrent stimulations with LPS, while microglia decrease (“tolerate”) production of pro-inflammatory mediators (TNF-α, IL-1β, and PGE_2_), they instigate (“sensitize”) the synthesis of anti-inflammatory mediators (IL-10) [11]. In other words, LPS-tolerized microglia become refractory to a subsequent endotoxin challenge referred to as a neuroprotective mechanism, targeted at the prevention of excessive toxic damage from cytokine production. Accordingly, activated microglia are the main target for alleviating neuroinflammation, including immunotolerance and low-grade inflammation, in order to prevent pathogenesis of various neurological and psychiatric disorders [12,13,14]. Noteworthily, the presence of other brain cells, such as neurons and astroglia, regulates the endotoxin tolerance capacity of microglia in the TNF-α reduction and IL-10 enhancement through M-CSF-mediated ERK signals [11]. In fact, little is known about how other brain cells interact with microglia in shaping their innate immune memory.

Inducible cyclooxygenase-2 (COX-2) catalyzes the first committed step in the synthesis of PGE_2_ and subsequently activates its downstream signaling pathways through four E-prostanoid (EP) receptors [15]. Activating PGE_2_ signals contributes to the neurotoxic effect of COX-2 in a broad spectrum of neurological disease models in the CNS [16]—from models of cerebral ischemia [17] to models of neurodegeneration and inflammation [18,19]. In addition to the high neural COX-2 activity in acute paradigms of excitotoxicity [20] (e.g., cerebral ischemia [21] and seizures [22]), microglia also show an increase in COX-2 activity and PGE_2_ production, causing inflammatory injury in inflammatory paradigms [10,23,24], such as Alzheimer’s disease [24,25], Parkinson’s disease [26], and amyotrophic lateral sclerosis [27]. Thus, the COX-2/PGE_2_ axis plays an important role in promoting neuronal injury, both in acute excitotoxic insults and in chronic neurodegenerative diseases [15,16,19]. Nevertheless, regulatory mechanisms for immune suppression (tolerance) of the COX-2/PGE_2_ axis in the brain are still unclear. The purpose of this study is to determine the tolerance mechanism of microglial PGE_2_ in response to repeated LPS challenges.

## 2. Materials and Methods

### 2.1. Animals

Pregnant C57/6J mice (*n* = 18) and their pups (*n* = 55) were purchased from the National Laboratory Animal Center (NLAC) in Tainan, Taiwan. Housing and breeding of the animals were performed humanely and with regard to alleviating suffering following the National Institutes of Health Guide for Care and Use of Laboratory Animals (Institute of Laboratory Animal Resources 1996). All procedures were approved by the National Cheng Kung University (NCKU) Animal Care and Use Committee.

### 2.2. Reagents

LPS (*E. coli* O111:B4, Cat# 437627, protein contaminants ≤ 2.0%, nucleic acid contaminants ≤ 2.5%) was obtained from EMD Chemicals, Inc. (Darmstadt, Germany). Recombinant TLR4 binding protein and cycloheximide were purchased from R&D Systems (Minneapolis, MN, USA) and Sigma-Aldrich (Saint Louis, MO, USA), respectively. Formaldehyde solution was obtained from Sigma-Aldrich (Saint Louis, MO, USA).

### 2.3. Preparation of Primary Neuron–Glia, Mixed Glia, and Microglia- and Astrocyte-Enriched Cultures

**Figure 1:**
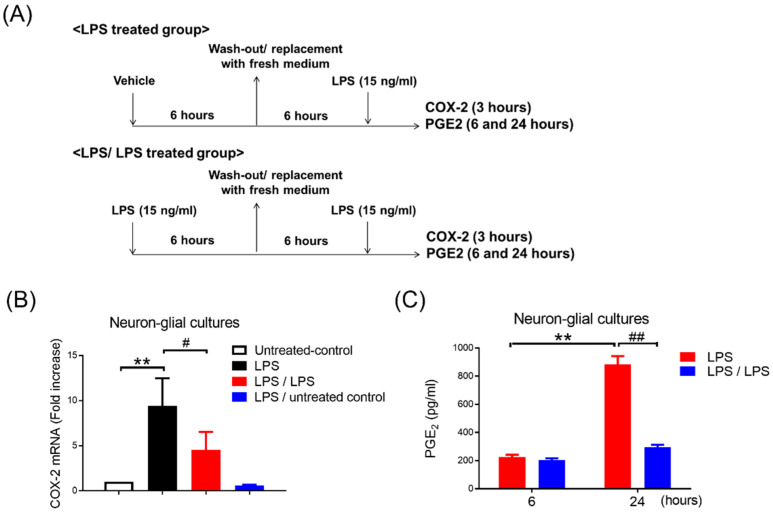
Reduction in COX-2 and PGE_2_ expression in neuron–glial cultures in response to endotoxin tolerance. (**A**) Experimental procedure for the study of PGE_2_ production in endotoxin tolerance. Neuron–glial (NG) cultures prepared from E14.5 time-pregnant C56/6J mice were pre-treated with vehicle (LPS-treated group) or LPS (15 ng/mL) (LPS/LPS-treated group) for 6 h. These pre-treated NG cultures were replaced with fresh media. Six hours later, LPS (15 ng/mL) was added to these NG cells. The level of COX-2 gene expression and supernatant PGE_2_ production was measured at 3, 6, and 24 h after LPS treatment. (**B**) After 3 h, the mRNA level of the COX-2 gene was measured in these NG cultures with untreated control, LPS-treated group (LPS), LPS/LPS-treated group (LPS/LPS), and LPS-untreated control group by RT-PCR. Three independent experiments were performed in duplicate. Data are expressed as a percentage of the LPS group (mean ± SEM). ** *p* < 0.01 vs. untreated control; # *p* < 0.05 vs. LPS. (**C**) A supernatant level of PGE_2_ in these cells with LPS-treated (LPS) and LPS/LPS-treated group (LPS/LPS) was detected at 6 and 24 h after LPS treatment by ELISA. Data are expressed as the mean ± SEM from three independent experiments in duplicate, ** *p* < 0.01 vs. 6H, ## *p* < 0.01 vs. LPS.

The preparation of mesencephalic neuron–glia cultures was performed from the mesencephalon of embryos at gestation day 14 ± 0.5 of the C57/6J mice (*n* = 18), as previously reported [10,11]. Briefly, after dissection and dissociation of mesencephalic tissues with mild mechanical trituration, cells were seeded to 24-well (5 × 10^5^ cells/well) culture plates precoated with poly-D-lysine (20 μg/mL) and maintained in 0.5 mL/well of MEM medium (10% heat-inactivated fetal bovine serum (FBS), 10% heat-inactivated horse serum (HS), 1 g/L glucose, 2 mM L-glutamine, 1 mM sodium pyruvate, and 0.1 mM nonessential amino acids). Cultures were preserved at 37 °C in a humidified atmosphere of 5% CO_2_/95% air. Three days later, 0.5 mL/well of fresh medium was replenished into the cultures. Seven days after seeding, the neuron–glia cultures made up of about 10% microglia, 50% astrocytes, and 40% neurons based on the visual counting of immunostained cells with antibodies against cell-type specific markers: neurons (Neu-N), microglia (OX-42), and astrocytes (GFAP) [28]. The NG cultures were ready for further endotoxin tolerance treatment regimen (Figure 1A). The neuron-enriched culture contained 99% neurons and less than 1% glia. The dividing glia was depleted from neuron–glia cultures 48 h after seeding with 8–10 μM of cytosine β-d-arabinofuranoside (Ara-C; Sigma-Aldrich, St. Louis, MO, USA) for three days.

Primary mixed glia cultures were prepared from whole brains of postnatal day-1 pups (*n* = 10) from the C57BL/6J mice [10,11]. After brain tissue disassociation, the cells were seeded onto 6-well (1 × 10^6^ cells/well) culture plates and maintained in 1 mL/well of DMEM/F-12 medium (10% FBS, 2 mM of L-glutamine, 1 mM of sodium pyruvate, and 0.1 mM of nonessential amino acids). Before reaching confluence, the medium was changed every 3 days. The mixed glia cultures contained about 80% astrocytes and 20% microglia and were used for endotoxin tolerance treatment regimen.

Microglia-enriched cultures were prepared from the whole brains of 1-day-old C57/6J pups (*n* = 45), as previously reported [10,11]. Briefly, after the dissociation of brain tissues, devoid of meninges and blood vessels by mild mechanical trituration, the isolated cells (5 × 10^7^ cells) were seeded in 150 cm^2^ culture flasks in DMEM/F12 medium (10% FBS, 2 mM of L-glutamine, 1 mM of sodium pyruvate, 0.1 mM of nonessential amino acids, 50 U/mL of penicillin, and 50 μg/mL of streptomycin) and maintained at 37 °C in a humidified atmosphere of 5% CO_2_/95% air. Before reaching confluence, the medium was changed 4 days later. Upon reaching confluence (12–14 days), the enriched microglia (99% pure) were obtained by shaking the flasks for 60 min at 180 rpm.

### 2.4. Cell Treatment

Multiple reconstituted brain cultures, including neuron–glial (NG), mixed glial (MG), microglia-enriched, fixed neurons plus microglia, and neurons plus microglia in Transwell inserts, were pre-incubated with or without LPS (15 ng/mL) for 6 h. After replacing the fresh media and waiting for an additional 6 h, LPS was readded into these cells (Figure 1A). Thus, endotoxin tolerance (ET) treatment regimen included untreated control, LPS (LPS alone treatment), LPS/LPS (twice LPS treatment), and LPS-untreated control groups. The expressions of COX-2 or PGE_2_ were measured at 3, 6, and 24 h in these cells by RT-PCR and ELISA, respectively. Furthermore, serum-free medium (no LPS binding protein (LBP)) and the addition of LBP (1 μg/mL) were used to study TLR4′s role in the development of microglial PGE_2_ tolerance in NG cells. Moreover, treated NG cells with cycloheximide, an inhibitor for protein synthesis, was performed to determine involvement of de novo protein synthesis in PGE_2_ tolerance of microglia.

### 2.5. Quantitative Real Time-PCR

According to the manufacturer’s instruction, the RNeasy Mini Kit (QIAGEN, Valencia, CA, USA) and the MuLV reverse transcriptase (Applied Biosystems, Foster City, CA, USA) were used to isolate the total cellular RNA of cells and synthesize the first-strand cDNA. After reverse transcription reaction, the SYBR-Green Master Mix (Applied Biosystems, Foster City, CA, USA) was used to perform real-time quantitative PCR analysis with the following PCR conditions: hold at 95 °C for 10 min and start 40 cycles at 95 °C for 15 s and 60 °C for 1 min. Data were normalized to a GAPDH expression. Vector NTI Advance 11.5 software (Invitrogen, Waltham, MA, USA) was used to design the primers. The sequences of the primers were the following: mouse COX-2 forward primer 5′ -TGA-TAT-GTC-TTC-CAG-CCC-ATT G- 3′; mouse COX-2 reverse primer 5′ -AAC-GGA-ACT-AAG-AGG-AGC-AGC- 3′; mouse GAPDH forward primer 5′ -TTC-AAC-GGC-ACA-GTC-AAG-GC- 3′; mouse GAPDH reverse primer 5′ -GAC-TCC-ACG-ACA-TAC-TCA-GCA-CC- 3′.

### 2.6. Measurement of PGE_2_

PGE_2_ in the culture medium was measured with the commercial ELISA kits from R&D Systems (Minneapolis, MN, USA).

### 2.7. Statistical Analysis

All data are expressed as the mean ± standard error of mean (SEM) and were compared between groups using the Student’s *t* test, as well as one-way or two-way analysis of variance (ANOVA) with Bonferroni’s multiple comparisons test (Prism 7; GraphPad Software, San Diego, CA, USA). A *p* value of <0.05 was considered statistically significant. *: *p* < 0.05; **: *p* < 0.01; ***: *p* < 0.001.

## 3. Results

To determine whether endotoxin tolerance (ET) of a microglial COX-2-PGE_2_ axis occurred, the ET treatment regimen (as described in Section 2.4; Figure 1A) was performed in primary neuron–glial (NG) cultures, containing 40% neurons, 50% astroglia, and 10% microglia. The expressions of COX-2 mRNA and supernatant PGE_2_ were measured at 3, 6, and 24 h in the NG cells by RT-PCR and ELISA, respectively. RT-PCR data showed that the LPS treatment induced mRNA levels of the COX-2 gene in the NG cells (1 vs. 9.41 ± 1.25, *p* < 0.01, one-way ANOVA, Figure 1B). Conversely, the NG cells received with 6 h LPS pre-incubation had decreased the expression of the subsequent endotoxin-induced COX-2 mRNA by 50% (9.41 ± 1.24 vs. 4.55 ± 0.81, *p* < 0.05, one-way ANOVA, Figure 1B). Our data indicated that the refectory to up-regulation of COX-2 mRNA occurred in the ET-treated NG cells (Figure 1B). The NG cells with a treatment regimen of saline (untreated control) or the LPS plus untreated control had no effect on the COX-2 induction (Figure 1B). Furthermore, ELISA data revealed that the production of PGE2 was induced in the supernatant of the LPS-treated NG cells at 24 h (225 ± 16.86 ng/mL vs. 883.67 ± 58.03 ng/mL, *p* < 0.01, two-way ANOVA, Figure 1C). Similar to the expression profile of the COX-2 mRNA, the NG cells with LPS pre-incubation had lower PGE_2_ production following subsequent LPS treatment (LPS/LPS) at 24 h in comparison with the NG cells with LPS alone treatment (LPS) (883.67 ± 58.03 ng/mL vs. 294 ± 19.15 ng/mL, *p* < 0.01, two-way ANOVA, Figure 1C). Accordingly, our data indicated that microglia were capable of programing COX-2-PGE_2_ axis tolerance in NG cells.

**Figure 2:**
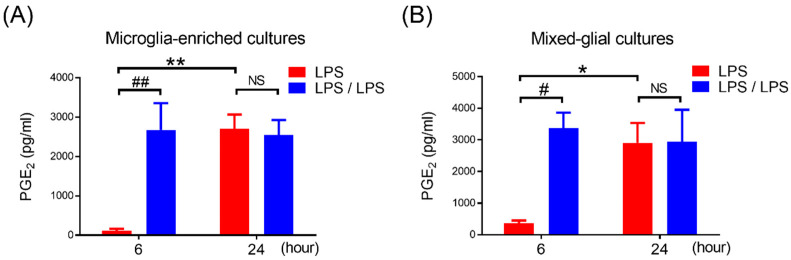
Failure of PGE_2_ reduction in microglia-enriched and mixed glia cells with endotoxin tolerance challenge. (**A**,**B**) A supernatant level of PGE_2_ in microglia-enriched cultures (**A**) and mixed glia cultures (MG) containing 80% astroglia and 20% microglia (**B**) in LPS-treated and LPS/LPS-treated regimen were detected at 6 and 24 h after treatment by ELISA. Five independent experiments were performed in duplicate. Data are expressed as the mean ± SEM, * *p* < 0.05, ** *p* < 0.01 vs. 6H; # *p* < 0.05, ## *p* < 0.01 vs. LPS. NS: no significant differences.

Under the LPS challenge, microglia are the main resource of the brain in producing PGE_2_. Then, we determined if the development of PGE_2_ reduction also occurred in microglia during the ET challenge. Microglia-enriched cultures were prepared and subjected to the same ET treatment regimen, shown in Figure 1A. Our data showed that the production of PGE_2_ in LPS pre-treated microglia (LPS/LPS group) was significantly increased in comparison with the microglia without LPS pre-treatment (LPS group) at 6 h of endotoxin treatment (116.6 ± 46.98 ng/mL vs. 2674.6 ± 680.35 ng/mL, *p* < 0.01, two-way ANOVA, Figure 2A). Meanwhile, similar to the microglia with once LPS treatment, the microglia with LPS pre-treatment produced a certain amount of PGE_2_ production at 24 h of endotoxin treatment (2701.2 ± 364.94 ng/mL vs. 2540.2 ± 386.34 ng/mL, Figure 2A). These results suggested that microglia alone failed to develop PGE_2_ tolerance during the ET challenge. Furthermore, to determine whether astroglia played a role in PGE_2_ reduction in tolerant microglia, the mixed glial cultures containing microglia and astroglia were prepared and applied to the same ET experimental procedure (Figure 1A). Our data revealed that compared to the cells with LPS treatment (LPS group), the pre-treatment of mixed glial (MG) cells with LPS (LPS/LPS group) increased the production of PGE_2_ at 6 h (374 ± 78.9 ng/mL vs. 3365.6 ± 495.66 ng/mL, *p* < 0.05, two-way ANOVA, Figure 2B) and failed to show PGE_2_ reduction at 24 h after endotoxin treatment (2910.4 ± 624.88 ng/mL vs. 2942.2 ± 1008.49 ng/mL, Figure 2B). The expression profile of PGE_2_ in microglia-enriched cultures and MG cells during endotoxin tolerance were similar (Figure 2). In other words, the presence of astroglia was unable to program PGE_2_ reduction in tolerant microglia. 

**Figure 3:**
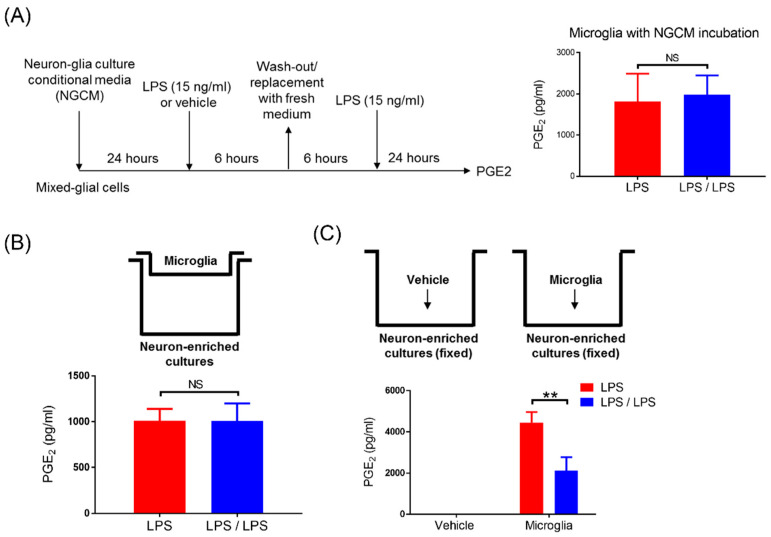
Neural contacts reversed the failure of PGE_2_ tolerance in microglia. (**A**) After 24 h incubation with neuron–glial condition media (NGCM), mixed glia (MG) cultures were subjected to the LPS-treated and LPS/LPS-treated regimen, as indicated in the right panel. PGE_2_ production in the supernatant of these treated MG cells after 24 h of treatment was measured by ELISA. Data are expressed as the mean ± SEM from three independent experiments in duplicate. NS: no significant differences. (**B**) Microglia were added into Transwell inserts while neurons grew confluent in the lower compartment of the 24-well plate, as indicated in the upper panel. After 24 h of incubation, these cells were applied to the LPS-treated and LPS/LPS-treated regimen. A supernatant level of PGE_2_ in these treated cells was detected by ELISA at 24 h of treatment. Data are expressed as the mean ± SEM from three independent experiments in duplicate. NS: no significant differences. (**C**) As indicated in the upper panels, the fixed neuron-enriched cultures were prepared and added with or without microglia. After 24 h of incubation, microglia were applied to the LPS-treated and LPS/LPS-treated regimen. A supernatant level of PGE_2_ in these treated microglia was measured by ELISA 24 h after treatment. Three independent experiments were performed in duplicate. Data are expressed as the mean ± SEM, LPS group versus the LPS/LPS group. ** *p* < 0.01. NS: not significant -.

According to Figure 1 and Figure 2, while PGE_2_ tolerance occurred in NG cultures, it did not occur in microglia-enriched and MG cultures, implying that the presence of neurons may participate in PGE_2_ reduction in tolerant microglia. We further determined whether soluble factors were secreted by neuron-regulated, tolerant microglia for PGE_2_ reduction. Thus, the condition media from neuron–glial cells (NGCM) were collected and added into the mixed glial cultures (Figure 3A). After 24 h of incubation, these MG cells were applied to the same ET treatment regimen (Figure 1A). Our data revealed that the incubation of MG cells with NGCM failed to restore the tolerant capacity of microglia in PGE_2_ reduction (1822 ± 388.5 ng/mL vs. 1984 ± 268 ng/mL, *p* = 0.74, Student’s *t*-test, Figure 3A). Alternatively, by using the Transwell culture system, the microglia in the upper inserts had no direct cell–cell contacts with neurons grown in the lower compartment of the culture plate (Figure 3B, upper panel). However, soluble factors were permeable between the plate’s upper and lower compartments (Figure 3B, upper panel). Further, our data showed that the production of PGE_2_ in these microglia with either once LPS (LPS group) or twice LPS treatment (LPS/LPS group) were comparable (1010 ± 75.35 ng/mL vs. 1006 ± 112 ng/mL, *p* = 0.97, Student’s *t*-test, Figure 3B, bottom panel), suggesting that neural soluble factors were not sufficient for PGE_2_ reduction in tolerant microglia. Subsequently, we examined whether physical contact with neurons was involved in PGE_2_ reduction in tolerant microglia. Neuron-enriched cultures were fixed with 4% formaldehyde solution and washed out with PBS three times. Although the fixed, dead neurons were unable to produce any soluble factors, they still presented antigen on their cell surface. Microglia were added into the fixed neurons for 24 h of incubation (Figure 3C, upper panel) and applied to the same ET treatment regimen (Figure 1A). Our data showed that PGE_2_ reduction occurred in microglia with fixed neurons in response to the ET treatment (4450.66 ± 297.37 ng/mL vs. 2125.33 ± 375.36 ng/mL, *p* < 0.01, two-way ANOVA, Figure 3C, bottom panel). Fixed neurons had no effect on PGE_2_ production (Figure 3C, bottom panel). In other words, the loss of PGE_2_ tolerance in microglia alone was recovered when it contacted with neurons (Figure 2A and Figure 3C). Moreover, our data indicated that the neuron–microglia contacts were critically involved in the development of the microglial ET capacity on PGE_2_ reduction.

**Figure 4:**
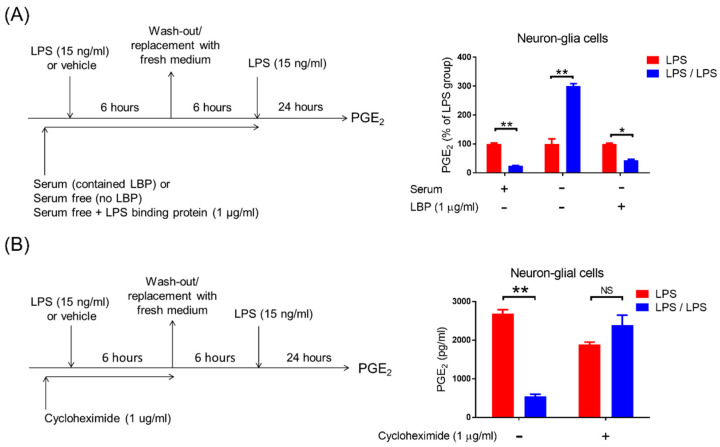
TLR4 signal and de novo protein synthesis is required for the development of PGE_2_ tolerance in neuron–glial cells. (**A**) Left panel: Experimental procedure for studying the TLR4’s role in PGE_2_ reduction during the endotoxin tolerance challenges. Right panel: Measurement of PGE_2_ production in the NG cultures with or without endotoxin tolerance challenge (LPS-treated versus LPS/LPS-treated group) in the conditions of 10% serum medium, serum-free medium, or serum-free plus LBP (1 μg/mL) at 24 h by ELISA. Data are expressed as the mean ± SEM from three independent experiments in duplicate, LPS group versus the LPS/LPS group. * *p* < 0.05, ** *p* < 0.01. (**B**) Left panel: Experimental procedure for studying the role of de novo protein synthesis in PGE_2_ reduction in response to endotoxin tolerance. Right panel: After treatment with cycloheximide (1 μg/mL) for 1 h, the NG cultures were applied to the procedure of the endotoxin tolerance challenge (LPS-treated versus LPS/LPS-treated group). PGE_2_ level in the supernatant was detected 24 h after treatment by ELISA. Data are expressed as the mean ± SEM from three independent experiments in duplicate, LPS group versus the LPS/LPS group. ** *p* < 0.01, NS, not significant.

Previous studies demonstrate that the activation of toll-like receptor 4 (TLR4) by LPS is critical for downstream inflammatory [29,30], anti-inflammatory [10], and tolerance responses [31]. Thus, we determined whether the TLR4-derived signal participated in the modulation of microglial PGE_2_ tolerance by neurons. Due to LPS-contained hydrophobic multi-acyl chains forming aggregates or micelles in aqueous solutions, the accessory LPS-binding proteins (LBPs) are required to mediate the sensitive recognition of LPS as well as their efficient transfer to the TLR4 [32,33]. After binding to LPS, the TLR4 signaling cascades are activated in the host immune response [30]. Therefore, by using serum-free medium (no LBP) with or without addition of recombinant LBP protein to incubate NG cells, the role of TLR4 signal in PGE tolerance was studied (Figure 4A, left panel). Our data revealed that during the ET treatment, PGE_2_ reduction occurred in NG cells at 24 h in the presence of serum medium-contained LBP (100 ± 3.11 vs. 23.79 ± 1.35, *p* < 0.01, two-way ANOVA, Figure 4A, right panel). Conversely, in serum-free media (no LBP), PGE_2_ tolerance disappeared (even higher PGE_2_ production) in NG cells at 24 h during the ET challenge (100 ± 17.43 ng/mL vs. 300.53 ± 8.15 ng/mL, *p* < 0.01, two-way ANOVA, Figure 4A, right panel). Furthermore, a recombinant LBP protein was added into serum-free media of NG cultures to confirm whether the TLR4 signal activation was crucial for the development of PGE_2_ tolerance. Our results revealed that adding recombinant LBP protein at 1 μg/mL concentration entirely reversed the failure of PGE_2_ tolerance in NG cultures at a serum-free condition (100 ± 2.4 ng/mL vs. 44.02 ± 2.75 ng/mL, *p* < 0.01, two-way ANOVA, Figure 4A, right panel). In addition, our data indicated that the TLR4-derived signal is necessary for PGE_2_ tolerance in microglia. Moreover, to determine whether inducing de novo protein synthesis by TLR4 activation was required for programming PGE_2_ tolerance, NG cells were treated with a protein synthesis inhibitor cycloheximide at 1 μg/mL concentration and subsequently applied to the same ET treatment regimen (Figure 4B, left panel). Our data showed that the inhibition of protein synthesis by cycloheximide disrupted the PGE_2_ tolerance in NG cells during ET (1882.66 ± 67.62 ng/mL vs. 2394.33 ± 252.02 ng/mL, Figure 4B, right panel). Moreover, our results suggested that the TLR4-dependent de novo protein synthesis participated in neuron-mediated PGE_2_ tolerance in microglia.

## 4. Discussion

**Figure 5:**
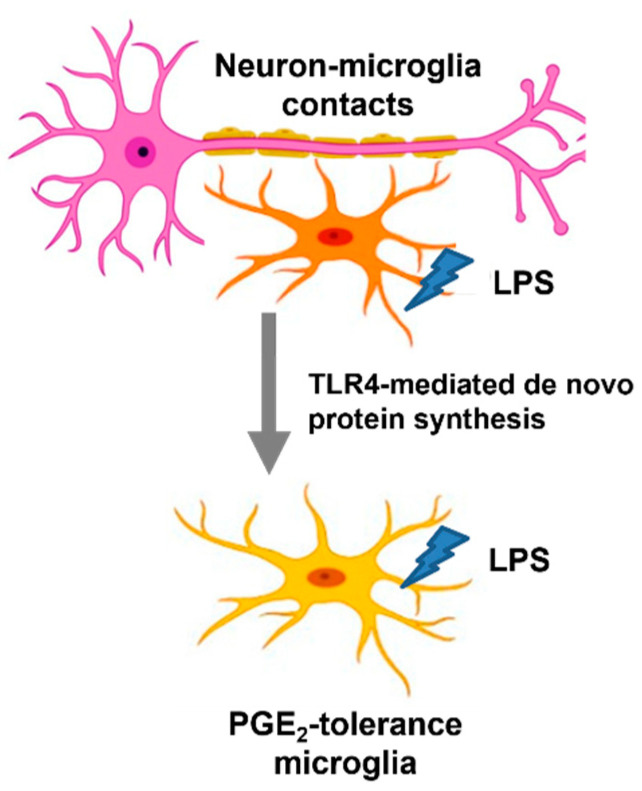
Indispensable role of neuron–microglia contacts for PGE_2_ tolerance via TLR4-dependant de novo protein synthesis. Schematic of neuron–microglia contacts alter the immune property of microglia for development of PGE_2_ tolerance via TLR4-derived de novo protein synthesis under ET challenge. Without neural contacts, microglia alone or cultured with astroglia or incubated with neural soluble factors fail to show endotoxin tolerance of PGE_2_.

As the first responder to the immune challenge, microglia secrete a wide spectrum and various inflammatory factors at inflammatory conditions, including IL-1β, TNF-α, PGE_2_, and BDNF, to prevent invading pathogens [34]. However, the uncontrolled and unresolved inflammation induced by microglia can damage the neurons [34]. It is relatively difficult to distinguish the functional role of microglia as either “protective” or “injurious” to the neurons during the neuroinflammatory process. Having a better understanding of heterogenous microglial activation during the inflammatory process, such as the occurrence of microglial endotoxin tolerance, has become a critical issue in developing microglia-based therapy for inflammation-related brain diseases [35,36]. Through using multiple reconstituted brain cell cultures, including neuron–glial, mixed glial, neuron-enriched, and microglia-enriched cultures, the main strength of the current study is to uncover the regulatory mechanisms of microglial PGE_2_ tolerance by interacting with other brain cells, such as neurons and astroglia. However, this NG culture system does not contain oligodendrocytes, which are the myelinating cells of the CNS. Interestingly, endotoxin tolerance of PGE_2_ occurs in NG cells (Figure 1), implying that oligodendrocytes do not participate in the regulation of PGE_2_ tolerance in microglia. Together, this study explored the immune-suppressive mechanism of PGE_2_ production mediated by neuron–microglia interactions via TLR4 signal-derived de novo protein synthesis in response to repeated LPS exposure (Figure 5).

In addition to electrical signal transmission, neurons are important immune regulators in restraining immune activation of homeostatic microglia at normal conditions, referred to as immune checkpoint [37,38]. The communications among neurons and microglia are bidirectional and reciprocal through various soluble factors and in receptor–ligand interactions [38,39]. With a volume transmission manner, neurons release the soluble factors out of the synaptic cleft to trigger receptor-mediated signals in microglia [40,41]. The neural soluble factors, such as ATP, glutamate, GABA, CSF-1, and TGF-β, are capable of regulating phagocytosis, motility, and viability of microglia [40,42,43]. On the other hand, many receptor ligands (i.e., CD47, CD200, CD22, and HSP60) on the surface of neurons directly bind with their corresponding surface receptors on microglia (i.e., CD172a, CD200R, CD45, and TREM2) that represent the classical contact-dependent communications [44,45,46,47,48]. Overall, these humoral or contacts signals from neurons not only lead microglia to prune neural synapses and neurites, and remove the apoptotic neurons during early brain development [45,47], but they also modulate motility, surveillance, and immunity of microglia at inflammatory conditions [44,46,48]. Our data showed that, in response to the ET challenge, microglial PGE_2_ tolerance occurred in the presence of neurons (Figure 1), while microglia alone or microglia co-cultured with astroglia failed to develop PGE_2_ tolerance (Figure 2). Furthermore, neuron–microglia contacts participate in neuron-mediated PGE_2_ tolerance in microglia (Figure 3). Receptor–ligand interactions among neurons and microglia may exert their functions to control microglial PGE_2_ tolerance. However, molecular details in neural contacts for microglia ET development remain an open question that will be further investigated.

Toll-like receptors (TLRs) function as the prime cellular sensors in innate immune cells for microbial components. Thus, its activation must be properly controlled by various mechanisms to maintain homeostasis. For instance, the induction of endotoxin tolerance by TLR4-ligand lipopolysaccharide (LPS) is one mechanism to prevent overstimulation from continuous exposure to the same and related danger signals [49]. The activation of the LPS receptor complex induces TLR4 dimerization/oligomerization with rapid activation of the MyD88-dependent signaling and TRIF-dependent signaling pathway, and further triggers various transcription factors, leading to strong production of pro-inflammatory cytokines [50]. Additionally, the activity of the microRNA miR-146a—known to target key elements of the myeloid differentiation factor 88 (MyD88) signaling pathway—including IL-1 receptor-associated kinase (IRAK1), IRAK2, and tumor necrosis factor (TNF) receptor-associated factor 6 (TRAF6), has been reported to establish and sustain tolerance [51]. Our data revealed that TLR4 activation and de novo protein synthesis are required for developing neuron govern PGE_2_ tolerance in microglia during ET (Figure 4). It is important to further study the mechanisms underlying neurons that modulate microglial TLR4 activation, its downstream signaling pathways, and de novo protein synthesis to preserve PGE_2_ tolerance.

Although the mechanism of ET formation in the brain and cultured brain slices or microglial cells have been reported [52,53,54,55,56], microglial PGE_2_ tolerance has not been fully investigated. Dr. Ajmone-Cat and his colleagues have been the first to report that the production of TNF-α, nitric oxide (NO), PGE_2_, and 15-deoxy-Δ12,14-PGJ2 (15d-PGJ2) was measured in primary rat microglial cultures received to one, two, or three consecutive LPS stimulations [53]. The results indicated that the ability of microglial cells to produce NO, TNF-α, and 15d-PGJ2 upon the first LPS challenge rapidly declined after the second and third stimulations, whereas cyclooxygenase-2 and PGE_2_ synthesis remained constantly elevated [53]. Further mechanistic studies in the transcription factors nuclear factor kappa B and CREB and the p38 MAPK revealed that the single or multiple LPS stimulations evoke profoundly different signaling pathways [53]. Even if the ET treatment regimens and species are distinct, similar results in this study also showed the failure of PGE_2_ tolerance (even having higher PGE_2_ production) in mouse microglia-enriched cultures with repeated LPS exposure (Figure 2). Accordingly, these data suggested that the circumstance of the CNS microenvironment, such as the presence of healthy neurons, plays an important regulating role in developing microglial ET [11]. Alternatively, they can determine if neurodegeneration-associated molecular patterns (NAMPs) participate in the disruption of microglial ET and whether its mechanism may provide attracted immune therapeutic targets for neurodegenerative disease.

In this study, we identified a distinct and essential role of non-immune brain cells, i.e., neurons in the development of PGE_2_ tolerance in microglia. In the absence of neurons, microglia-enriched and mixed glial cultures failed to form PGE_2_ tolerance. Notably, neural contacts program microglial PGE_2_ tolerance—not its soluble factors. To the best of our knowledge, our study provides the first evidence that non-immune cells, i.e., neurons, can influence the capacity of microglial PGE_2_. Moreover, this study revealed a novel regulatory role of neuron–microglia contacts in the development of microglial PGE_2_.

## Data Availability

All relevant data are within the paper.
